# Supplementary material for: The prioritisation of curable sexually transmitted infections among pregnant women in Zambia and Papua New Guinea: Qualitative insights
Source: PLOS Glob Public Health. 2024 Jul 25;4(7):e0003441. doi: 10.1371/journal.pgph.0003441 (PMC11290942; doi:10.1371/journal.pgph.0003441)
Supplement: Attachment — Submitted filename: ResponsetoCommentsonmanuscript_9Apr2024.docx [file pgph.0003441.s002.docx]

**Manuscript number** PGPH-D-23-01976

**Full title (original)** The prioritisation of curable sexually transmitted infections among pregnant women Africa and the Pacific: Qualitative insights from health care workers, policy makers and programme advisors in Zambia and PNG.

**Corresponding Author** Angela Kelly-Hanku

This paper is informative and explores an important issue but require major revisions. Below are my suggestions and comments for the authors:

Many thanks for the important feedback on our paper. Please see below our responses to your queries. We have numbered these for ease of cross-referencing. Where we have changed the text, we give the line numbers of the new text in the clean version of the revised manuscript.

| - **TITLE**   1. The title of the manuscript is very lengthy and confusing. So, it needs to change the title to make short and easy understandable. The title may be renamed as “The prioritisation of curable sexually transmitted infections among pregnant women in Zambia and Papua New Guinea: Qualitative insights” | Thank you, we have condensed the title to the following:  The prioritisation of curable sexually transmitted infections among pregnant women in Zambia and Papua New Guinea: Qualitative insights |
| --- | --- |
| - **INTRODUCTION**   2. This section well apprises on curable STIs and the little attention paid to these infections (except HIV). However, this section lacks information on the study’s research question, i.e. how the STIs are prioritized among expecting women who are under antenatal care. Information on this issue are available at global level; the authors are recommended to provide more information so as to better portray the need to explore the research question. | Many thanks for highlighting this. We have developed our introduction to further respond to this comment. Please see p4, lines 82-87  *At a country level there is some indication of the prioritisation of STIs, with many countries adopting updated STI treatment guidelines and the monitoring of gonococcal antimicrobial resistance (1). Despite these indications of prioritisation, other indicators, such as aetiological testing of STIs, is still far from the norm in LMIC settings which have the greatest burden of these curable STIs. Outside of the “triple elimination agenda, it is unknown how, if at all, curable STIs are being prioritised locally among pregnant women in antenatal care.* |
| 3. It is written that the two countries, namely Zambia and Papua New Guinea were selected due to different socio-cultural and epidemiological settings. However, there is no mention of how this difference contributes to this research. Recommended to elaborate in this regard. | *Thank you. We have revised this paragraph in the manuscript to clarify our reason for selecting these sites. The paragraph now reads (p4, lines 88-98):*  *Against this backdrop of prioritisation of STIs in maternal and child health, and as part of a larger multidisciplinary study on the political prioritisation of STIs(2), we used the platforms of two clinical trials, one in Zambia (3) and the other in Papua New Guinea (PNG) (4). These two clinical trials were, in whole or in part, investigating the role of STIs in pregnancy and adverse maternal and newborn health outcomes. Our study aimed to understand ways in which STIs in pregnancy are prioritised, understood and addressed at the local level These countries were chosen because of our ongoing research in these two countries. In both study sites the prevalence of curable STI among pregnant women attending antenatal clinics is high, around 35% in Zambia (5) and 43% in PNG (6). In contrast, Zambia is considered a high HIV burden country with HIV prevalence among adults 15-49 years estimated at almost 11% whereas in PNG, HIV prevalence is low, at 1% for the same population (7), but as high as 20% among female sex workers (11-20%) (8).* |
| - **METHODS**   4. Lines 106-108: Interviews were conducted……………. were severely affected. Interviews were conducted with whom? How many respondents were? Please write one or two sentence about it and refer to Table 1. | Thank you. We have added the total number of health care workers and key informants, 19 in total in the first line of the second paragraph in methods section and include, broadly who the informants were. (p5, lines 105-114).  *Semi-structured interviews were conducted with 19 health care workers and key informants to explore the treatment and care of pregnant women with STIs, socio-cultural issues and other factors affecting the diagnosis and treatment of STIs in pregnant women, and the impacts of prioritisation of STIs in pregnancy.* Semi-structured interviews are qualitative in nature and for research with key informants this method was deemed appropriate as it allowed us to enquire about specific issues but allowed the opportunity for the informants and interviewers to address other issues as they emerged. Semi structured interview guides were used for all the interviews. *Health care workers included those working in rural and district health facilities in provincial and urban settings in the study sites in the two provinces (one on Zambia, one in PNG). Key informants included senior technical advisers working in policy and programme management (table 1).*  We have also added a line regarding the use of semi-structured interviews as an appropriate method for this research. |
| 5. Please include the information on study respondents in this section. It is unclear whether the respondents are from rural or urban areas and the types of facilities that the health care workers were employed at. There are examples of health service delivery differing according to geographical areas and different types of health facilities. | Thank you for highlighting this. In addition to the response above we have added additional information. It now reads: (p6, lines 132-140):  *Participants were purposively selected from clinical, public health and sexual reproductive and health technical advisory groups to provide detailed emic accounts of STIs in pregnancy. Participants included those working at district and rural health facilities at our study sites. Key informants were selected based on their specialist knowledge relating to maternal health and/ or STI programs, working at the national level. In both PNG and Zambia interviews were conducted until saturation was met, whereby no more new information was being heard. Across the two study sites we conducted interviews health care workers, key informants and women attending for antenatal care. In this paper, we report on data derived from semi-structured interviews conducted with health care workers and key informants.* |
| 6. Semi-structured interviews. Please make clear about it. Face to face interview? Or in-depth interview…………….? | We have attended to this, please see comment #4 above. |
| 7. The weakest area of the paper is the methods section. Without elaborating about following aspects, it is difficult to evaluate the paper: | Thank you for this comment. We have responded to each issue raised below. We have also indicated where the issue has been addressed in comments 1-6. |
| 8. Why the two countries- Zambia and PNG were selected? Please provide detail information. | Many thanks, we have now included more detail on why these 2 countries were included in our study. This has been updated, in response to comment 3, and now reads as included below. The major changes are highlighted with red font below: (page 4; lines 88-98)  *Against this backdrop of prioritisation of STIs in maternal and child health, and as part of a larger multidisciplinary study on the political prioritisation of STIs (9), we used the platforms of two clinical trials that examined STIs in pregnancy, one in Zambia (3) and the other in Papua New Guinea (PNG) (4).* These two clinical trials were, in whole or in part, investigating the role of STIs in pregnancy and adverse maternal and newborn health outcomes.  *These countries were chosen due to our ongoing research in these two countries. In both study sites the prevalence of curable STI among pregnant women attending antenatal clinics is high, around 35% in Zambia (5) and 43% in PNG (6). In contrast, Zambia is considered a high HIV burden country with HIV prevalence among adults 15-49 years estimated at almost 11% whereas in PNG, HIV prevalence is low, at 1% for the same population (7), but as high as 20% among female sex workers (11-20%) (8).* |
| 9. Purposively selected only 19 participants. What were the selection processes? Nothing has mentioned about this in the paper. | We have included more detail on how and why the 19 participants were recruited and this is now covered in query # 4&5 above. |
| 10. The respondents were selected from which levels? i.e. national, regional, district, sub-district levels. | Many thanks, we hope we have now covered this query in our responses to comments #4, 5 and 9. |
| 11. How the sample size was determined from different levels. Please provide detail information. | We have added the following detail in the methods section to explain the sample size. It now reads: (p6; lines 136-137)  *In both PNG and Zambia interviews were conducted until data saturation was met, whereby no more new information was emerging.* |
| 12. Interview guides were co-designed by the lead researchers in Zambia and PNG. What does it mean? Please provide information related to the guidelines used for data collection, i.e. details about the guidelines, type of guidelines developed and used for data collection with different type of respondents? Were the guidelines pre-tested before collecting data? | We have included a line and, where relevant included the initials of the leads for the qualitative leads for this study. Online training took place due to COVID-19 travel restrictions.  Please see p 6; lines 119-122  Interview *guides were designed by the lead researchers in Zambia and PNG (AKH, KK). Teams of experienced and highly skilled qualitative researchers from the Tropical Diseases Research Centre, Ndola, Zambia and the PNG Institute of Medical Research were trained together via online platforms (AKH, KK), owing to COVID-19 travel restrictions, as well as face-to-face training sessions in each country in preparation for data collection.*  We have included a line to explain the development and refinement of the interview guides (p 6, lines 123-125)  *Interview guides were refined by the team throughout the training and during the initial stages of interviews with ongoing support and were discussion between the investigators and the qualitative field team.* |
| 13. Semi-structured interviews were conducted with healthcare workers? But the study title shows that it is a qualitative study. How those matched? Why other methods of qualitative data collection were not applied for data collection which might allow the authors to do data triangulation? | Thank you for this comment. We would like to clarify that semi-structured interviews are a particular type of qualitative interview.  Please see response to query #4 above.  Semi-structured interviews allow for pre-defined areas for questioning, while also allowing flexibility to explore other issues as they emerge.  Whilst it is recognised by most qualitative researchers that semi-structured interviews are qualitative, in case some readers might use the term for a quantitative research method. We have made this clearer through the addition of a sentence describing qualitative research in this context (p 5; lines 108-110)  At the design phase of this study, the team discussed the research methods and decided that the research question would be best addressed through semi-structured interviews. |
| 14. Please provide information about the data collectors, training, data quality control . | The qualitative interviewers were experienced in the conduct of in-depth interviews, in addition they received additional training for this study.  Please see our response to comment 12 and p5; lines 119-125 |
| 15. How data quality were controlled at different stages? | We believe this is now covered in comment #12, above.  All recordings were transcribed at each site, with transcription taking place as soon as possible after the interview. We have amended to the following: (p 6; lines 128-131)  *In each country audio recordings were transcribed and, as necessary, translated into English for analysis and then stored, managed and coded in NVivo v12. Translation was conducted by experienced qualitative researchers in the respective countries. All translations were cross-checked for quality and accuracy.* |
| 16. Key informants were interviews, who were the key informants? Were healthcare workers also key informants? | Thank you for seeking this clarification. We hope this is now clearer, following our response to comment 4 and as included on page 5; lines 112-116, where it now reads:  *Health care workers included those working in rural and district health facilities in provincial and urban settings. Key informants included senior technical advisers working in policy and program management.* |
| 17. The sample size is not ample enough to cover the situation of two countries. Population in both Zambia and Papua New Guinea stands at millions whereas only a total of 19 respondents were interviewed from both the countries. It is recommended that the authors shrink down the study areas to regional or local levels according to where the respondents are employed at. | Thank you for this remark. We appreciate the overall population size of each of the countries.  Please see response to query #5 above.  As a qualitative study we do not seek to have population level representation as is the case in quantitative research. Instead, we have sought to understand in-depth the overall sense of the prioritization of STIs in these respective countries. For example, in UN agencies such as UNFPA there is usually only one designated maternal health specifically for the country and they are, by virtue of their role, able to speak across the country. We interviewed health care workers in the local areas in each country where the clinical trials were being implemented. This ensured we could speak to both the national and local level perspectives. We have now clarified this further in the methods section, please see p6; lines 133-137.  *Participants were purposively selected from clinical, public health and sexual reproductive and health technical advisory groups to provide detailed emic accounts of STIs in pregnancy. Participants included those working at district and rural health facilities at our study sites. Key informants were selected based on their specialist knowledge relating to maternal health and/ or STI programs, working at the national level.* |
| - **RESULTS**   18. The result section focuses more on challenges, under each theme instead of providing a concrete overview on STIs prioritization among expecting women under antenatal care. It is recommended that they provide a balanced write up. It is also recommended to include some information related to existing policy and knowledge of respondents on existing policies in this regard. | We appreciate the concern of the reviewer and the need for a balanced write up of study results. However, based on the data, we have reported what the participants discussed – they spoke of a lack of prioritisation through the lens of the challenges to address STIs for pregnant women.    The issue of policy is an interesting one and was part of our larger project. We have added reference x, a policy analysis, which was published after we submitted this article for review. We did not seek to determine people’s knowledge of global or national policy but rather understand practices. The issue of knowledge is an interesting one, and one we may seek to explore in the future using quantitative approaches of a representative sample of health care workers. Other research in the larger study examined global policy, I include a reference to this work.  [Understanding the factors affecting global political priority for controlling sexually transmitted infections: a qualitative policy analysis - PMC (nih.gov)](https://www.ncbi.nlm.nih.gov/pmc/articles/PMC10823925/) |
| 19. It is good that the results are presented following WHO building blocks, but the presentation could be made more logically. The authors are advised to rewrite the section focusing the key variables comparing between the two countries. A table can be provided with the key common findings in the two countries for easy understanding of the readers. | Thank you for recognising the presenting of our findings against the WHO building blocks.  We recognize that there are different ways that a qualitative study of this kind could be written, including, for example comparing two countries against each other. Given our focus was not one of comparing the countries against each other, but of how priorit isation is happening in practice using PNG and Zambia as case studies, we have considered the reviewers comments but feel it remains important to think about the health system and prioritisation more broadly. Whilst tables can be useful in qualitative research, we feel that a table comparing the 2 countries wouldn’t strengthen the narrative of our findings. |
| - **DISCUSSION**   20. This section appears to be a summarized version of the result section. The authors are requested to re-write this section and focus more on the interpretations and implications derived from their study findings. This will help the authors to portray the significance of their results in successfully addressing their research question. | Many thanks for your comments regarding our discussion. We have reviewed and revised the discussion section and included additional work to discuss the implication of our findings, rather than summarizing what we found. Please see pages 21-25. |
| - **OTHER REMARKS**   21. The writing is comprehensible but requires improvements. It is recommended that the manuscript be revised for language and grammar. | Thank you, we have reviewed this manuscript again for language and grammar. |

References

1.	Unemo M, Lahra MM, Escher M, Eremin S, Cole MJ, Galarza P, et al. WHO global antimicrobial resistance surveillance for Neisseria gonorrhoeae 2017-18: a retrospective observational study. Lancet Microbe. 2021;2(11):e627-e36.

2.	Wu D, Low N, Hawkes SJ. Understanding the factors affecting global political priority for controlling sexually transmitted infections: a qualitative policy analysis. BMJ Glob Health. 2024;9(1).

3.	Chico R.M, Chandramohan D. The ASPIRE Trial - Aiming for Safe Pregnancies by Reducing Malaria and Infections of the Reproductive Tract <https://clinicaltrials.gov/ct2/show/NCT04189744> 2019 [

4.	Vallely AJ, Pomat WS, Homer C, Guy R, Luchters S, Mola GDL, et al. Point-of-care testing and treatment of sexually transmitted infections to improve birth outcomes in high-burden, low-income settings: Study protocol for a cluster randomized crossover trial (the WANTAIM Trial, Papua New Guinea). Wellcome Open Res. 2019;4:53.

5.	Chaponda EB, Chico RM, Bruce J, Michelo C, Vwalika B, Mharakurwa S, et al. Malarial Infection and Curable Sexually Transmitted and Reproductive Tract Infections Among Pregnant Women in a Rural District of Zambia. The American journal of tropical medicine and hygiene. 2016;95(5):1069-76.

6.	Vallely LM, Toliman P, Ryan C, Rai G, Wapling J, Tomado C, et al. Prevalence and risk factors of Chlamydia trachomatis, Neisseria gonorrhoeae, Trichomonas vaginalis and other sexually transmissible infections among women attending antenatal clinics in three provinces in Papua New Guinea: a cross-sectional survey. Sex Health. 2016;13(5):420-7.

7.	UNAIDS. UNAIDS Data 2022 <https://www.unaids.org/sites/default/files/media_asset/data-book-2022_en.pdf>. 2023.

8.	Kelly-Hanku A, Weikum D, Badman SG, Willie B, Boli-Neo R, Kupul M, et al. Factors associated with HIV and syphilis infection among female sex workers in three cities in Papua New Guinea: findings from Kauntim mi tu, a biobehavioral survey. Sex Health. 2020;17(4):311-20.

9.	Swiss Network of International Studies. Political Prioritisation of the Prevention and Control of Sexually Transmitted Infections: a Global Challenge [Available from: <https://snis.ch/project/political-prioritisation-of-the-prevention-and-control-of-sexually-transmitted-infections-a-global-challenge/>.
